# Supplementary material for: Iterative improvement in the automatic modular design of robot swarms
Source: PeerJ Comput Sci. 2020 Dec 7;6:e322. doi: 10.7717/peerj-cs.322 (PMC7924708; doi:10.7717/peerj-cs.322)
Supplement: Supplemental Information 3 [file peerj-cs-06-322-s003.zip › argos3/doc/api/standalone/a00474_source.html]

ARGoS: plugins/robots/foot-bot/simulator/footbot\_turret\_default\_actuator.cpp Source File


- Main Page
- Related Pages
- Namespaces
- Classes
- Files

- File List
- File Members

# plugins/robots/foot-bot/simulator/footbot\_turret\_default\_actuator.cpp

Go to the documentation of this file.

```
00001 
00007 #include "footbot_turret_default_actuator.h"
00008 
00009 namespace argos {
00010 
00011          const Real RPM_TO_RADIANS_PER_SEC = ARGOS_PI / 30.0f;
00012 
00013    /****************************************/
00014    /****************************************/
00015 
00016    CFootBotTurretDefaultActuator::CFootBotTurretDefaultActuator() :
00017       m_pcTurretEntity(NULL),
00018       m_unDesiredMode(CFootBotTurretEntity::MODE_OFF) {}
00019 
00020    /****************************************/
00021    /****************************************/
00022 
00023    void CFootBotTurretDefaultActuator::SetRobot(CComposableEntity& c_entity) {
00024       m_pcTurretEntity = &(c_entity.GetComponent<CFootBotTurretEntity>("turret"));
00025       m_pcTurretEntity->Enable();
00026    }
00027 
00028    /****************************************/
00029    /****************************************/
00030 
00031    void CFootBotTurretDefaultActuator::SetRotation(const CRadians& c_angle) {
00032       m_pcTurretEntity->SetDesiredRotation(c_angle);
00033    }
00034 
00035    /****************************************/
00036    /****************************************/
00037 
00038    void CFootBotTurretDefaultActuator::SetRotationSpeed(SInt32 n_speed_pulses) {
00039       m_pcTurretEntity->SetDesiredRotationSpeed(RPM_TO_RADIANS_PER_SEC * n_speed_pulses);
00040    }
00041 
00042    /****************************************/
00043    /****************************************/
00044 
00045    void CFootBotTurretDefaultActuator::SetMode(ETurretModes e_mode) {
00046       m_unDesiredMode = e_mode;
00047    }
00048 
00049    /****************************************/
00050    /****************************************/
00051 
00052    void CFootBotTurretDefaultActuator::Update() {
00053       m_pcTurretEntity->SetMode(m_unDesiredMode);
00054    }
00055 
00056    /****************************************/
00057    /****************************************/
00058 
00059    void CFootBotTurretDefaultActuator::Reset() {
00060       m_unDesiredMode = CFootBotTurretEntity::MODE_OFF;
00061    }
00062 
00063    /****************************************/
00064    /****************************************/
00065 
00066    REGISTER_ACTUATOR(CFootBotTurretDefaultActuator,
00067                      "footbot_turret", "default",
00068                      "Carlo Pinciroli [ilpincy@gmail.com]",
00069                      "1.0",
00070                      "The foot-bot turret actuator.",
00071                      "This actuator controls the foot-bot turret. For a complete\n"
00072                      "description of its usage, refer to the ci_footbot_turret_actuator\n"
00073                      "file.\n\n"
00074                      "REQUIRED XML CONFIGURATION\n\n"
00075                      "  <controllers>\n"
00076                      "    ...\n"
00077                      "    <my_controller ...>\n"
00078                      "      ...\n"
00079                      "      <actuators>\n"
00080                      "        ...\n"
00081                      "        <footbot_turret implementation=\"default\" />\n"
00082                      "        ...\n"
00083                      "      </actuators>\n"
00084                      "      ...\n"
00085                      "    </my_controller>\n"
00086                      "    ...\n"
00087                      "  </controllers>\n\n"
00088                      "OPTIONAL XML CONFIGURATION\n\n"
00089                      "None for the time being.\n",
00090                      "Usable"
00091       );
00092 
00093 }
```

---

Generated on 10 Jul 2018 for ARGoS by 
 1.6.1
